# MLH1 AND MSH2 PROTEIN EXPRESSION LOSS AND MICROSATELLITE INSTABILITY IN COLORECTAL ADENOMAS OF PATIENTS WITH LYNCH SYNDROME: A SYSTEMATIC REVIEW AND META-ANALYSIS

**DOI:** 10.1590/S0004-2803.24612026-081

**Published:** 2026-10-30

**Authors:** Gustavo Carreira XAVIER, Lucas Araújo VAZ, Marina Cota CALDEIRA, Hacer TEKIN, Tarak DHAOUADI

**Affiliations:** 1 Federal University of Viçosa, Medicine and Nursing Department, Viçosa, MG, Brazil.; 2 Faculty Ciências Médicas de Minas Gerais, Medicine Department, Belo Horizonte, MG, Brazil.; 3 Gaziantep University, Faculty of Medicine, Şahinbey/Gaziantep, Turkey.; 4 Tunis El Manar University, Charles Nicolle Hospital, Research Laboratory in Immunology of Renal Transplantation and Immunopathology (LR03SP01), Tunisia.

**Keywords:** Microsatellite instability, adenoma, lynch syndrome, systematic review, meta-analysis, Instabilidade de microssatélites, adenoma, síndrome de lynch, revisão sistemática, metanálise

## Abstract

**Background:**

Lynch Syndrome (LS) is a genetic condition associated with a high risk of colorectal cancer, often linked to microsatellite instability (MSI), which is caused by defects in DNA mismatch repair (MMR) genes such as MLH1 and MSH2. Although MMR deficiency and MSI are well-documented in the literature, the role of these genes in adenomas remains unclear.

**Objectives:**

To evaluate, through meta-analysis, the correlation between loss of expression of the MLH1 and MSH2 genes and MSI in adenomas from patients with LS.

**Methods:**

A systematic search was conducted in the MEDLINE, SCOPUS, and Web of Science databases up to 06/09/2025, using a strategy with descriptors related to MSI, LS, and precursor lesions. Data were analyzed using the Inverse Variance method, with 95% confidence intervals. Heterogeneity was assessed using the I-squared ( I²) statistic. Outliers were identified through statistical influence diagnostics applied to each study. Publication bias was assessed using funnel plots and Egger’s regression test. Statistical analyses were performed using RStudio, version 4.4.0.

**Results:**

5 studies were included, totaling 145 adenomas from individuals with LS. A highly significant correlation was found between MSI and MSH2 expression loss (OR=13,91 [3,97-48,72]; *P*=0,000038; I²=0%). No significant correlation was found between MSI and loss of MLH1 expression (OR=2,19 [0,93-5,16]; *P*=0,073; I²=0%). Funnel plot analysis and Egger’s regression test showed no significant evidence of publication bias for MLH1 (t=0.25, df=3, *P*-value=0.8155) or MSH2 (t=-0.79, df=3, *P*-value=0.4860).

**Conclusion:**

This meta-analysis found a strong, significant and consistent correlation between MSI and MSH2 expression loss in adenomas, but a non-significant association for MLH1 expression loss. The lack of statistical significance in Egger’s test for both genes should be interpreted with caution due to the small number of included studies (k=5). The limited literature available highlights the need for further, larger-scale research.

## HIGHLIGHTS

• The study aimed to evaluate, through a meta-analysis, the correlation between the loss of MLH1 and MSH2 gene expression and microsatellite instability (MSI) in adenomas from patients with Lynch Syndrome.

• A systematic search was conducted in MEDLINE, SCOPUS, and Web of Science databases up to September 2025, including 5 studies totaling 145 adenomas analyzed using the Inverse Variance method.

• The findings revealed a highly significant correlation between MSI and MSH2 expression loss (OR=13.91), whereas the association for MLH1 loss did not reach statistical significance (OR=2.19).

• The meta-analysis concluded that MSH2 loss is a consistent marker of MSI in adenomas, suggesting that different mismatch repair genes may contribute unequally to early tumorigenesis in Lynch Syndrome.

## RESUMO

**Contexto:**

A Síndrome de Lynch (SL) é uma condição genética associada a um alto risco de câncer colorretal, frequentemente ligada à instabilidade de microssatélites (IMS), que é causada por defeitos nos genes do sistema de reparo de incompatibilidade de DNA (MMR), como MLH1 e MSH2. Embora a deficiência de MMR e a IMS sejam bem documentadas na literatura, o papel desses genes em adenomas permanece incerto.

**Objetivo:**

Avaliar, por meio de meta-análise, a correlação entre a perda de expressão dos genes MLH1 e MSH2 e a IMS em adenomas de pacientes com SL.

**Métodos:**

Uma busca sistemática foi conduzida nas bases de dados MEDLINE, SCOPUS e Web of Science até 06/09/2025, utilizando uma estratégia com descritores relacionados a IMS, SL e lesões precursoras. Os dados foram analisados utilizando o método da Variância Inversa, com intervalos de confiança de 95%. A heterogeneidade foi avaliada utilizando a estatística I-quadrado (I²). Outliers foram identificados por meio de diagnósticos de influência estatística aplicados a cada estudo. O viés de publicação foi avaliado utilizando gráficos de funil e o teste de regressão de Egger. As análises estatísticas foram realizadas utilizando o RStudio, versão 4.4.0.

**Resultados:**

Foram incluídos 5 estudos, totalizando 145 adenomas de indivíduos com SL. Uma correlação altamente significativa foi encontrada entre IMS e perda de expressão de MSH2 (OR=13,91 [3,97-48,72]; *P*=0,000038; I²=0%). Nenhuma correlação significativa foi encontrada entre IMS e perda de expressão de MLH1 (OR=2,19 [0,93-5,16]; *P*=0,073; I²=0%). A análise do gráfico de funil e o teste de regressão de Egger não mostraram evidências significativas de viés de publicação para MLH1 (t=0,25, df=3, *P*-valor=0,8155) ou MSH2 (t=-0,79, df=3, *P*-valor=0,4860).

**Conclusão:**

Esta meta-análise encontrou uma correlação forte, significativa e consistente entre IMS e perda de expressão de MSH2 em adenomas, mas uma associação não significativa para a perda de expressão de MLH1. A falta de significância estatística no teste de Egger para ambos os genes deve ser interpretada com cautela devido ao pequeno número de estudos incluídos (k=5). A literatura limitada disponível destaca a necessidade de pesquisas futuras em maior escala.

## INTRODUCTION

Lynch Syndrome (LS), one of the most prevalent hereditary cancer syndromes, is an autosomal dominant condition that confers a markedly increased risk for colorectal cancer (CRC) as well as several extracolonic malignancies^1^ ^,^ ^2^. Advances in the understanding of its genetic basis and clinical implications have significantly shaped modern strategies for cancer surveillance, prevention, and management^3^ ^,^ ^4^.

At the core of LS pathogenesis are germline mutations in the DNA mismatch repair (MMR) system, primarily involving the MLH1 and MSH2 genes^1^ ^,^ ^3^. These genes play an essential role in maintaining genomic integrity by correcting replication errors that arise during DNA synthesis. Inactivation of MLH1 or MSH2 leads to MMR deficiency, resulting in the accumulation of mutations, particularly within short, repetitive DNA sequences known as microsatellites. This phenomenon, termed microsatellite instability (MSI), is a molecular hallmark of LS-associated tumors^5^. The assessment of MSI and the immunohistochemical detection of MLH1 and MSH2 protein loss have become pivotal diagnostic and prognostic tools for identifying LS carriers and for characterizing the molecular profile of their tumors^6^ ^,^ ^7^.

Although the strong correlation between MMR deficiency, MSI, and progression to CRC in LS patients is well established, the specific role of MLH1 and MSH2 protein loss and MSI in precursor lesions, such as colorectal adenomas, remains an area of active investigation^8^ ^,^ ^9^. Understanding these molecular dynamics at the premalignant stage is critical, since not all adenomas progress to carcinoma, and the identification of molecular markers of progression could refine surveillance strategies^10^.

Previous studies have shown that MMR deficiency is relatively common in adenomas arising in LS patients, and that analysis of MLH1 and MSH2 expression in these lesions may provide valuable insights into the early stages of tumorigenesis and into the risk of malignant transformation^9^ ^,^ ^11^. Moreover, emerging evidence suggests that the adenoma immune microenvironment, although more extensively characterized in sporadic cases, may also be influenced by MMR deficiency, potentially affecting lesion progression^12^.

Given the central role of MLH1 and MSH2 in the pathogenesis of LS and the need to clarify their involvement in adenomatous lesions, the present study aimed to evaluate, through meta-analysis, the correlation between loss of expression of these genes and MSI in adenomas from patients with LS. This investigation seeks to contribute to more precise risk stratification and to the development of improved clinical management strategies for individuals affected by LS.

## METHODS

### Search strategy

This study was performed according to the Preferred Reporting Items for Systematic Reviews and Meta-Analyses (PRISMA) guidelines for systematic reviews and meta-analyses^13^. An electronic literature search for eligible studies among all papers published prior to September 06, 2025, was conducted through PubMed, SCOPUS and Web of science databases. The following search string was used: (“microsatellite instability” OR “MSI-H” OR “DNA mismatch repair deficiency” OR “MMR-deficient”) AND (“Lynch syndrome” OR “hereditary nonpolyposis colorectal cancer” OR “HNPCC”) AND (“precancerous lesions” OR “colorectal adenoma” OR “colorectal polyp” OR “serrated lesion” OR “dysplasia”). The literature search was carried out without any language restriction.

### Selection criteria

All studies were independently assessed and evaluated by 2 reviewers (Gustavo Carreira Xavier, G.C.X. and Hacer Tekin, H.T.). The following selection criteria were adopted:

### Inclusion criteria

- Observational studies with cohort (prospective) or case-control (retrospective) design.
- Studies including patients with a confirmed diagnosis of Lynch Syndrome (via genetic testing or clinical criteria) presenting with colorectal adenomas.
- Studies evaluating loss of MLH1 and/or MSH2 expression (by immunohistochemistry or other validated methods) and/or MSI in adenomas.
- Peer-reviewed original research articles.

### Exclusion criteria

- Studies assessing only colorectal carcinomas or sporadic adenomas.
- Case reports, narrative reviews, systematic reviews, editorials, conference abstracts without sufficient data, animal studies, or experimental models.
- Studies with duplicate populations: in such cases, only the study with the largest sample size and/or most complete data was included.

### Definitions

The definition of LS was based on two criteria: subjects who have been unambiguously defined by a germline mutation, subjects with family history that fulfilled the Amsterdam I or II criteria^14^ ^,^ ^15^. MSI defined when exhibited, at least, one unstable loci or one band-shift at one of microsatellite markers, while microsatellite stable was defined when no MSI was found^16^. The presence of the proteins of the genes MLH1 and MSH2 was detected by immunohistochemistry. Proximal adenomas were located proximal to the splenic colon flexure, while distal adenomas were located distal to the same anatomical mark^17^.

### Data extraction

Data was extracted using a predeveloped form and entered in an Excel datasheet. Two investigators (G.C.X. and Lucas Araújo Vaz, L.A.V.) independently extracted the following information: reference, year of publication, patients, number of patients by sex (male and female), patient’s age at adenomas detection (mean and median), microsatellite status (adenomas with MSI and adenomas with microsatellite stability (MSS)) loss of MMR genes expression (MLH1, MSH2 and no mutations on MLH1 and/or MSH2), size of adenoma (<5, 5-10, >10 and no data) and location (proximal and distal) (Table 1). A third investigator (Marina Cota Caldeira, M.C.C.) compared the results of the extracted data for potential discrepancies.

**TABLE 1:** Study characteristics.

Study	Patients (n)	Sex		Patient’s age at adenomas detection		Microsatellite status			Loss MMR genes expression			Size of adenoma (mm)			
		Male (n)	Female (n)	Mean (years)	Median (years)	Number of adenomas (n)	Adenomas with MSI (n)	Adenomas with MSS (n)	MLH1 (n)	MSH2 (n)	None (n)	<5	5-10	>10	No data
Pino (2009)^14^	15	10	5	50,02	49	44	23	17	21	7	12	16	22	6	0
Halvarsson (2005)^17^	27	13	14	52,06	48	35	14	6	14	9	12	11	14	5	5
Giuffrè (2005)^19^	17	9	8	45,9	44	18	16	2	8	6	4	1	9	8	0
Iino (2000)^20^	24	10	14	-	-	30	24	6	4	14	6	14	9	7	0
Yurgelun (2012)^21^	21	11	10	49,65	49	37	18	19	7	10	20	22	11	2	2

*Ref: study reference. **n:** number of patients or adenomas. **MSI:** microsatellite instability. **MSS:** microsatellite stability. **MMR:** mismatch repair. **mm:** millimeters.

### Quality assessment

The methodological quality of the eligible studies was independently assessed by two reviewers (G.C.X. and L.A.V.) using the Joanna Briggs Institute Checklist for Analytical Cross-Sectional Studies, which evaluates key domains related to internal validity, including the clarity of inclusion criteria, appropriateness of sample and setting, validity and reliability of exposure and outcome measurements, identification and management of confounding factors, and adequacy of statistical analysis. Each study was appraised according to the nine Joanna Briggs Institute (JBI) criteria, and overall judgments were recorded as low, moderate, or high risk of bias based on the proportion of criteria met. Risk-of-bias assessments for each included study were additionally documented using a standardized form (Excel spreadsheet) and visualized through the Cochrane risk of bias visualization online tool (https://mcguinlu.shinyapps.io/robvis/). Two further independent reviewers (M.C.C. and Tarak Dhaouadi, T.D.) verified the quality assessment results. 

### Study endpoints

The primary endpoint of this meta-analysis was to determine the association between MSI and the loss of MLH1 and/or MSH2 expression in colorectal adenomas from patients with LS. Secondary endpoints included the evaluation of heterogeneity and potential sources of bias, assessed through outlier and influence analyses, as well as the investigation of publication bias using funnel plots and Egger’s regression test.

### Statistical analysis

Statistical analysis was carried out using RStudio, version 4.4.0. Pooled odds ratio (OR) with 95% confidence intervals (95%CI) were computed to assess the association between MSI and loss of MLH1 or MSH2 expression in adenomas from patients with LS. The significance of pooled OR was tested using a Z-test with a threshold of 0.05. Heterogeneity across studies was assessed using the I-squared ( I²) statistic. Random-effects models (Inverse Variance method) were applied as recommended by Borenstein et al.^13^. Given that the eligible studies were conducted in heterogeneous populations with differences in study design, sample size, and methods for assessing MSI and MMR protein expression, the use of a random-effects model was considered the most appropriate approach^13^ ^,^ ^18^. Outlier and influence diagnostics were performed to test the stability of the results by sequentially omitting each study. Forest plots were generated to illustrate the distribution of effect sizes across the included studies. Publication bias was evaluated using funnel plots and Egger’s regression test. Subsequently, heterogeneity was investigated by performing influence analyses, in which potential outliers were identified and sequentially omitted to assess their impact on the overall results. Outlier detection and evaluation were conducted using: 1) externally standardized residuals, 2) the difference-in-fit standardized (DFFITS,) statistic, 3) Cook’s distances, 4) covariance ratios, 5) leave-one-out tau-squared (Tau²) estimates, and 6) leave-one-out residual heterogeneity test statistics. Publication bias was assessed using Egger’s test and visualized with funnel plots. It should be noted that Egger’s regression requires at least three included studies.

A PRISMA checklist is available as a supplementary file.

### Systematic review registration

This review has been registered on PROSPERO: CRD420251142211, Available from: https://www.crd.york.ac.uk/PROSPERO/view/CRD420251142211

## RESULTS

### Search results and study characteristics

A PRISMA flow diagram was generated to illustrate the study selection process (Figure 1). Overall, 5 studies^14^ ^,^ ^17^ ^,^ ^19^ ^,^ ^20^ ^,^ ^21^ comprising a total of 145 adenomas from patients with LS were included in the present analysis. The characteristics of the included studies are summarized in TABLE 1. Notably, all 5 studies evaluated both the association between MLH1 loss and MSI and the association between MSH2 loss and MSI. Outlier and influence analyses were performed for both MLH1 and MSH2 datasets. Risks of bias for the included studies, assessed using the JBI Checklist for Analytical Cross-Sectional Stu­dies, are summarized in Figure 2.

**FIGURE 1:**
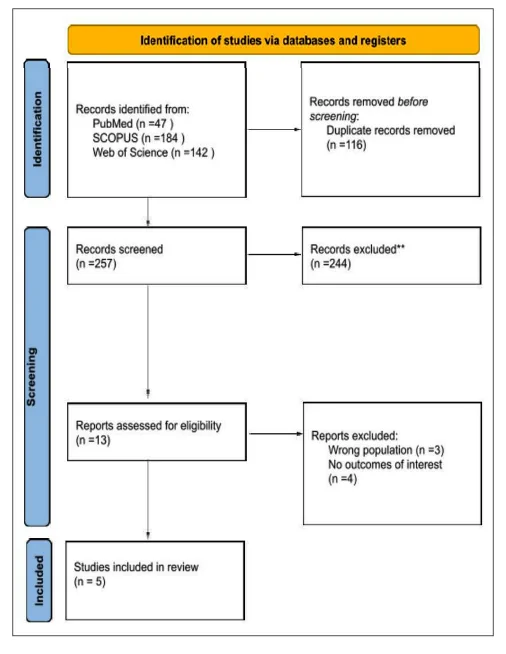
PRISMA flow diagram for study selection^18^.

**FIGURE 2:**
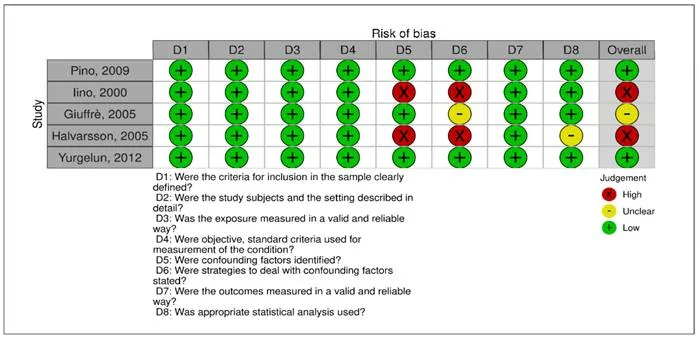
Summary of study risk of bias.

### MLH1 expression loss and microsatellite instability

Combined analysis showed no significant association between MLH1 loss and MSI in adenomas from LS patients, with an OR of 2.19 [0.93-5.16], *P*=0.07, 95% prediction interval (PI) = 0.65-7.38 (Table 2) (Figure 3). Heterogeneity between studies was null (I²=0%, *P*=0.57, Tau²=0). Outlier analysis revealed that the studies of Pino, 2009^14^ and Yurgelun, 2012^21^ were significantly outlying studies (Figure 4). Every outlier index (Externally standardized residuals, DFFITS statistic, Cook’s distances, Covariance ratios, Leave-one-out Tau^2^ estimates, Leave-one-out (residual) heterogeneity test statistic, Hat values and Study weight) showed that both these studies contributed significantly to the between-studies heterogeneity. However, their exclusion did not substantially alter the overall effect: OR=2.37 [0.46-12.25], *P*=0.30, PI=0.06-87.37 (Table 2) (Figure 5). The sensitivity analysis revealed that the association results were stable across the models (Figure 6), indicating a high level of robustness and reliability. In the analysis shown, no single study significantly altered the overall effect size or heterogeneity when omitted. This suggests that the final results are not driven by any individual study, strongly supporting the integrity and homogeneity of the findings.

**TABLE 2:** Loss of gene expression and microsatellite instability.

Expression Loss and Microsatellite Instability	OR [95CI]	***P*-value**	I^2^	***P*-value**	Tau^2^	95% PI
MLH1 expression loss and microsatellite instability	2.19 [0.93-5.16]	0.07	0%	0.57	0	0.65-7.38
Omitting Pino, 2009 and Yurgelun 2012	2.37 [0.46-12.25]	0.30	0%	0.43	0	0.06-87.37
MSH2 expression loss and microsatellite instability	OR=13.91 [3.97-48.72]	0.000038	0%	0.084	0	2.36-82.12

**FIGURE 3:**
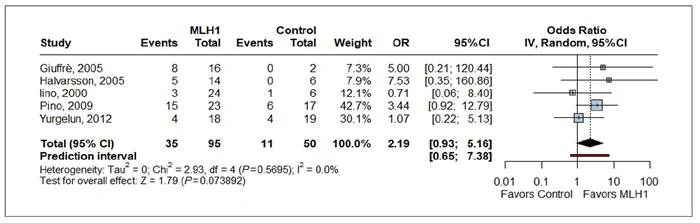
Forest plot for the association between the MLH1 expression loss and microsatellite instability.

**FIGURE 4:**
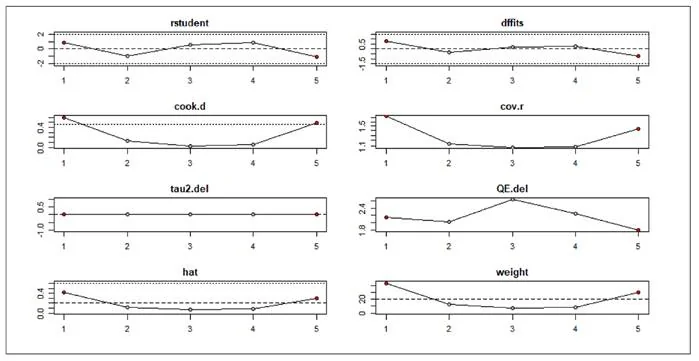
Outlier analysis for the association between the MLH1 expression loss and microsatellite instability. Externally standardized residuals, DFFITS statistic, Cook’s distances, Covariance ratios, Leave-one-out Tau^2^ estimates, Leave-one-out (residual) heterogeneity test statistic, Hat values and Study weight. **1:** Pino 2009, **2:** Iino 2000, **3:** Giuffrè 2005, **4:** Halvarsson 2005, **5:** Yurgelun 2012.

**FIGURE 5:**
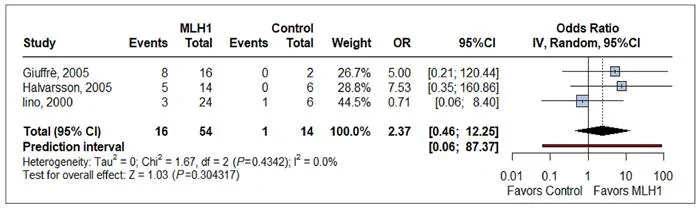
Forest plot for the association between the MLH1 expression loss and MSI omitting outlier studies.

**FIGURE 6:**
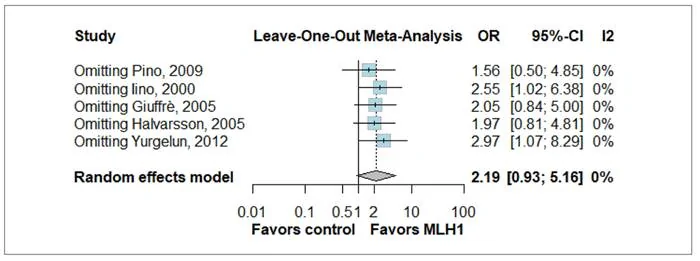
Sensitivity analysis from MLH1 data.

### MSH2 expression loss and microsatellite instability

For MSH2 analysis, there was a strong association between its loss and MSI in LS patients, with an OR of OR=13.91 [3.97-48.72], P=0.000038, I²=0%, Tau²=0, PI=2.36-82.12 (Figure 7). Between-study heterogeneity was moderate, with I²=49.3%, *P*=0.084, Tau²=0.61. No outliers were identified (Figure 8). No sensitivity analysis was conducted for MSH2, as the absence of heterogeneity (I²=0%) and influential outliers indicated a stable and homogeneous data set.

**FIGURE 7:**
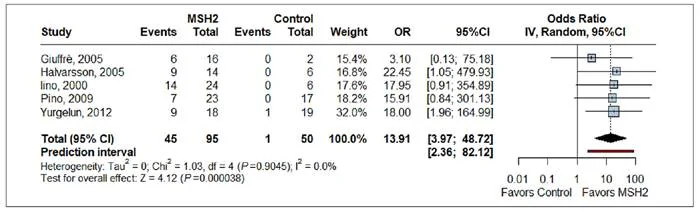
Forest plot for the association between the MSH2 expression loss and MSI.

**FIGURE 8:**
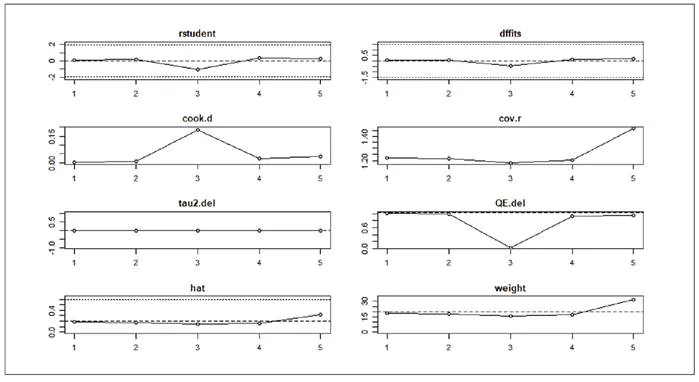
Outlier analysis for the association between the MSH2 expression loss and MSI. Externally standardized residuals, DFFITS statistic, Cook’s distances, Covariance ratios, Leave-one-out Tau^2^ estimates, Leave-one-out (residual) heterogeneity test statistic, Hat values and Study weight. **1:** Pino 2009, **2:** Iino 2000, **3:** Giuffrè 2005, **4:** Halvarsson 2005, **5:** Yurgelun 2012.

### Publication Bias

The funnel plot generated for MLH1 (Figure 9) appeared visually asymmetrical, suggesting a potential for publication bias or small-study effects. However, in contrast to this visual impression, Egger’s regression test yielded a non-significant result (t=0.25, df=3, *P*=0.8155), indicating that the findings of the meta-analysis were not substantially influenced by small-study effects or selective publication.

**FIGURE 9:**
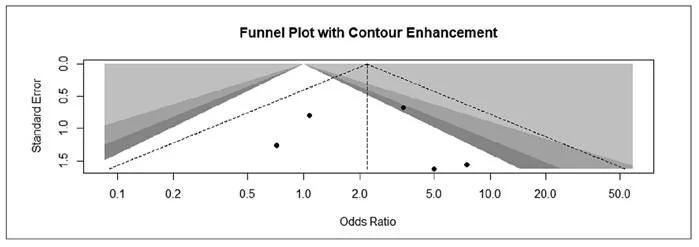
Funnel plot assessing publication bias in MLH1 data.

In contrast, the funnel plot for MSH2 (Figure 10) appeared visually symmetrical, particularly within the central cluster of higher-precision studies, suggesting a low likelihood of publication bias. Consistently, Egger’s test for MSH2 also showed a non-significant result (t=-0.79, df=3, *P*=0.4860), supporting the null hypothesis of symmetry.

**FIGURE 10:**
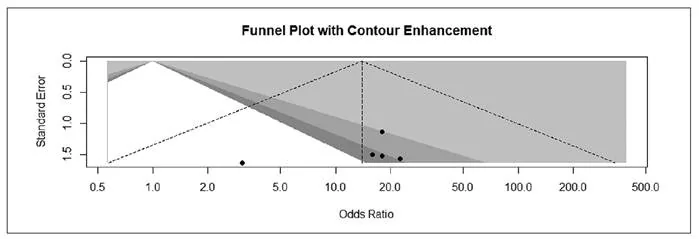
Funnel plots assessing publication bias in MSH2 data.

Nevertheless, the interpretation of funnel plots and Egger’s regression test should be approached with caution, as the small number of included studies (n=5) limits the statistical power to reliably detect potential publication bias.

Finally, there was a strong, consistent, and statistically significant association between MSH2 loss and MSI, while the association between MLH1 loss and MSI did not reach statistical significance. Both analyses demonstrated negligible heterogeneity and no evidence of publication bias, reinforcing the overall robustness and reliability of the findings.

## DISCUSSION

Our findings indicate that loss of MSH2 expression shows a strong and statistically significant association with MSI in adenomas arising in patients with LS, whereas loss of MLH1 expression does not demonstrate a statistically significant association with MSI (FIGURES 5 AND 7). This molecular divergence suggests that different MMR genes may contribute unequally to the early adenomatous phase of LS-associated tumorigenesis.

The strength of the association observed for MSH2 loss is remarkable both from a biological and a statistical standpoint. The pooled OR for MSH2 (OR=13.91; 95%CI: 3.97-48.72) indicates a magnitude of effect that is not only clinically meaningful but also highly stable across all included studies (Figure 7). Importantly, heterogeneity was negligible (I²=0%), and all leave-one-out analyses preserved both the statistical significance and the magnitude of the association, with OR estimates ranging narrowly from 12.32 to 18.30. The absence of influential points or outliers further reinforces the robustness of this finding. Taken together, the MSH2 results exhibit an exceptional degree of internal consistency, which strengthens the inference that MSH2 deficiency plays a dominant role in the initiation of MSI in LS-associated adenomas (FIGURE 8).

In contrast, the results for MLH1 demonstrate a more nuanced pattern. Although the pooled effect suggests a potential positive association (OR=2.19; 95%CI: 0.93-5.16), the CI includes the null value, and the p-value (0.07) falls just short of conventional significance thresholds (Figure 3). The leave-one-out sensitivity analyses revealed that the MLH1 meta-analytic estimate is more sensitive to individual study effects. When certain studies-such as those by Iino or Yurgelun-were omitted, the OR shifted toward statistical significance, while omission of others shifted the estimate in the opposite direction (Figure 5). This fluctuation indicates that MLH1-related findings are less stable and more dependent on individual sample characteristics than those observed for MSH2. While heterogeneity was low (I²=0%), the influence diagnostics identified two studies (Pino 2009 and Yurgelun 2012) as outliers in multiple statistical dimensions, despite the fact that their exclusion did not materially alter the pooled effect. This further illustrates that the available evidence for MLH1 is both limited and sensitive to study-level variability (Figure 4).

The strong and statistically consistent association between MSH2 loss and MSI aligns with the broader clinical phenotype observed in individuals with MSH2 mutations. Carriers of MSH2 germline variants not only exhibit a substantial risk of CRC but also a disproportionately elevated risk of urothelial carcinomas and cutaneous neoplasms, particularly those associated with the Muir-Torre variant of LS^22^. The stability of our meta-analytic findings supports the hypothesis that MSH2 deficiency may be an early and central event in MSI-driven adenoma formation in LS^23^.

This review also highlights notable methodological challenges within the existing literature. The included studies differ substantially in the criteria used to diagnose LS, ranging from germline-confirmed cases (Pino) to classic Amsterdam I/II (Giuffrè, Iino)^14^ ^,^ ^19^ ^,^ ^20^. Similarly, definitions of MSI varied widely, with studies using distinct instability thresholds, marker panels, and classification algorithms. These inconsistencies complicate cross-study comparability and may contribute to the relative instability of MLH1-related findings. Despite these limitations, our statistical analyses confirmed minimal heterogeneity for both genes and detected no evidence of publication bias through funnel plot inspection or Egger’s regression, although the small number of available studies inherently limits the diagnostic power of these tools (Figure 10).

Overall, this meta-analysis provides compelling evidence that MSH2 loss is strongly and consistently associated with MSI in LS-associated adenomas, while MLH1 loss, although suggestive of a similar direction of effect, lacks statistical stability and requires further investigation. Future research should prioritize standardized definitions of MSI, uniform immunohistochemical protocols for assessing MMR protein expression, and harmonized diagnostic criteria for LS. High-quality, adequately powered observational studies will be essential to clarify whether MLH1 loss contributes more subtly to early MSI development and to refine molecular markers for risk stratification and surveillance in LS.

## CONCLUSION

In conclusion, this meta-analysis provides robust evidence that MSH2 loss is strongly and consistently associated with MSI in adenomas from patients with LS, whereas the association between MLH1 loss and MSI was not statistically significant. The negligible heterogeneity and absence of publication bias across studies support the reliability and stability of these findings. Altogether, these results highlight the pivotal role of MSH2 deficiency in the early molecular events underlying MSI development in Lynch-associated adenomas and emphasize the need for further large-scale, well-designed studies to confirm and expand upon these observations.

## Data Availability

data in article: the research data are presented within the article itself (available in the Results section)
